# Circulating Galectin-1 and Galectin-3 in Sera From Patients With Systemic Sclerosis: Associations With Clinical Features and Treatment

**DOI:** 10.3389/fphar.2021.650605

**Published:** 2021-04-20

**Authors:** Victoria Sundblad, Ramiro A. Gomez, Juan C. Stupirski, Pablo F. Hockl, Maria S. Pino, Hugo Laborde, Gabriel A. Rabinovich

**Affiliations:** ^1^ Laboratorio de Inmunopatología, Instituto de Biología y Medicina Experimental (IBYME), Consejo Nacional de Investigaciones Científicas y Técnicas (CONICET), Ciudad de Buenos Aires, Argentina; ^2^ División Reumatología, Hospital de Clínicas “José de San Martín”, Universidad de Buenos Aires, Ciudad de Buenos Aires, Argentina; ^3^ Facultad de Ciencias Exactas y Naturales, Universidad de Buenos Aires, Ciudad de Buenos Aires, Argentina

**Keywords:** systemic sclerosis, galectin-1, galectin-3, inflammation, autoimmune diseases

## Abstract

Systemic Sclerosis (SSc) is a rheumatic disease characterized by fibrosis, microvascular damage and immune dysregulation. Two major subsets, limited cutaneous systemic sclerosis (lcSSc) and diffuse cutaneous systemic sclerosis (dcSSc) can be defined, according to the extent of skin involvement. Increasing evidence indicates a role for galectins in immune and vascular programs, extracellular matrix remodeling and fibrosis, suggesting their possible involvement in SSc. Here, we determined serum levels of galectin (Gal)-1 and Gal-3 in 83 SSc patients (dcSSc *n* = 17; lcSSc *n* = 64; ssSSc *n* = 2), and evaluated their association with clinical manifestations of the disease. Patients with dcSSc showed lower Gal-3 levels, compared to lcSSc (*p* = 0.003), whereas no considerable difference in Gal-1 levels was detected between groups. Remarkably, higher concentrations of Gal-1 were associated with the presence of telangiectasias (*p* = 0.015), and higher concentrations Gal-3 were associated with telangiectasias (*p* = 0.021), diarrhea (*p* = 0.039) and constipation (*p* = 0.038). Moreover, lower Gal-3 levels were associated with the presence of tendinous retractions (*p* = 0.005). Patients receiving calcium blockers (*p* = 0.048), methotrexate (*p* = 0.046) or any immunosuppressive treatment (*p* = 0.044) presented lower concentrations of Gal-3 compared to those not receiving such treatments. The presence of telangiectasia and the type of SSc maintained their statistical association with Gal-3 (*β* 0.25; *p* = 0.022 and *β* 0.26; *p* = 0.017, respectively) in multiple linear regression models. In conclusion, serum levels of Gal-3 are associated with clinical manifestations of SSc. Among them, the presence of telangiectasias could be explained by the central role of this lectin in the vascularization programs.

## Introduction

Systemic Sclerosis (SSc), also called scleroderma, is an immune-mediated rheumatic disease, characterized by microvascular damage and fibrosis. Although skin fibrosis is a typical hallmark in this progressive disease, patients present different patterns of organ-based complications. In fact, dysfunction and eventual failure of almost any internal organ can be observed (Allanore et al., 2015; Denton and Khanna, 2017). The considerable heterogeneity in the extent and severity of visceral organ commitment is indeed a major factor in determining SSc prognosis (Gabrielli et al., 2009). Most patients with SSc are classified into two major subsets, namely limited cutaneous systemic sclerosis (lcSSc) and diffuse cutaneous systemic sclerosis (dcSSc) subsets (LeRoy et al., 1988), according to the extent of skin involvement. While in lcSSc skin fibrosis is restricted to fingers (sclerodactyly), distal extremities and face, the trunk and proximal extremities are also affected in dcSSc. In addition, a small number of patients (<5%) present clinical manifestations (most commonly Raynaud’s phenomenon, digital ulcers, pulmonary arterial hypertension and scleroderma renal crisis) and serological features specific to systemic sclerosis, but in the absence of detectable skin involvement (i.e. systemic sclerosis sine scleroderma). Moreover, some patients show features of another connective tissue disease, such as rheumatoid arthritis, polymyositis or systemic lupus erythematosus, overlapping with systemic sclerosis. Even though the pathogenesis of SSc is complex and not completely understood, a distinctive triad of microvascular damage, dysregulation of innate and adaptive immunity, and generalized fibrosis in multiple organs characterizes this heterogeneous disease (LeRoy et al., 1988).

Galectins are a family of endogenous glycan-binding proteins characterized by a common structural fold and a conserved carbohydrate recognition domain (CRD) that recognizes glycans containing the disaccharide N-acetyllactosamine (Galβ1,4GlcNAc). These soluble proteins can function either in the extracellular milieu by interacting with a myriad of glycosylated receptors, or intracellularly by controlling signaling pathways through protein–glycan or protein–protein interactions (Cerliani et al., 2017). Several studies substantiate a role for galectin–glycan interactions in modulating the function of relevant cell surface receptors, thus modulating signaling pathways that govern immune and vascular programs, as well as extracellular matrix remodeling and fibrosis (Cerliani et al., 2017; Elola et al., 2018). Undoubtedly, the best studied members of the galectin family are the proto-type Galectin-1 (Gal-1) and the chimera-type galectin-3 (Gal-3) proteins, which are ubiquitously expressed in different tissues and display a broad range of biological functions. Widely expressed in inflammatory microenvironments, Gal-1 has emerged as a potent homeostatic signal that shapes immune responses, by targeting multiple cell types within the innate and adaptive immune compartments (Sundblad et al., 2017). On the other hand, Gal-3 exerts mostly pro-inflammatory effects by reinforcing activation of macrophages, dendritic cells (DCs), mast cells, and natural killer (NK) cells, as well as T and B lymphocytes (Sciacchitano et al., 2018), depending on its intracellular or extracellular localization and the implicated target cell type. Interestingly, increasing evidence shows that both Gal-1 and Gal-3 participate in vascular programs leading to development of blood vessel networks (Croci et al., 2014), and modulate fibroblast signaling programs, impacting on deposition of a cross-linked collagen matrix and fibrosis (Elola et al., 2018). Particularly Gal-3, promotes fibroblast proliferation and transformation, and mediates collagen production, modulating fibrogenesis in diverse organs, including liver, kidney, lung and myocardium (Li et al., 2014).

Given the involvement of Gal-1 and Gal-3 in angiogenesis, immunity and extracellular matrix remodeling, a role of these lectins in SSc development, characterized by vascular alterations, inflammation and fibrosis has been proposed. In this line, previous studies evaluated possible differences in Gal-1 (Yanaba et al., 2016) and Gal-3 (Taniguchi et al., 2012; Koca et al., 2014; Gruszewska et al., 2020) serum levels between SSc patients and controls, and reported different paradoxical results. In addition, possible associations between Gal-3 serum levels and SSc variants (Taniguchi et al., 2012; Stochmal et al., 2020), or between this lectin and specific clinical manifestations and laboratory markers of the disease has also been documented (Taniguchi et al., 2012; Koca et al., 2014; Yanaba et al., 2016; Faludi et al., 2017; Hromádka et al., 2017; Gruszewska et al., 2020; Stochmal et al., 2020), although with highly dissimilar results. Thus, further studies are required to reconcile these paradoxical different findings and to define a possible association between galectins and SSc progression.

Due to the remarkable heterogeneity in clinical disease signatures and to our limited understanding of the complex mechanisms underlying SSc development, the diagnosis and clinical management of patients with SSc is extremely challenging. Despite revised classification criteria, no scheme is completely useful to adequately capture the whole complexity and heterogeneity of this disease. Moreover, personalized assessment of disease manifestations, stratification of risk of future complications and individualized treatment of SS impose major challenges. New validated, non-invasive biomarkers are required to aid in the diagnosis, assessment of disease activity and response to therapeutic approaches in SSc (Wermuth et al., 2018). In line with this demand, and considering the previous inconclusive findings, we aimed at determining serum levels of Gal-1 and Gal-3 in patients with SSc and to evaluate possible associations with clinical manifestations of the disease and treatment options.

## Materials and Methods

### Patients

A descriptive, observational and cross-sectional study was conducted. Data were obtained retrospectively from the analysis of the electronic database of patients with SSc from the Rheumatology Service at the Hospital de Clínicas “José de San Martín”, University of Buenos Aires. Patients with a diagnosis of SSc, over 18 years of age, who met the ACR/EULAR 2013 criteria for SSc, with at least one visit to the Rheumatology Service between January 2008 and June 2018 and who had a serum sample in our library were included. Patients whose available data were not sufficient to meet the classification criteria, did not fulfill the definition of clinical variants of SSc, or did not have record of the date of diagnosis, clinical manifestations or determination of specific autoantibodies, were excluded. Patients were informed in detail about the study, and written consent was obtained in accordance with the Declaration of Helsinki. The protocol was approved by Ethics and Research Committees of Hospital de Clínicas “José de San Martín” and Instituto de Biología y Medicina Experimental (IBYME).

### Clinical Features

Gender, age and time of evolution of SSc (from diagnosis to galectin determination) were recorded. Clinical forms of the disease (dcSSc, lcSSc or *sine*SSc) were recorded according to LeRoy classification (LeRoy et al., 1988). The presence (current or past) of skin involvement (edematous phase or skin fibrosis), calcinosis, telangiectasias, arthritis (identified by rheumatologist), muscle weakness, Raynaud's phenomenon, interstitial lung disease (ILD) [defined by Computed Tomography resolution of the chest according to the definitions of the American Thoracic Society/European Respiratory Society 2002 consensus], pulmonary hypertension (PHT) (defined as PSAP ≥36 mmHg by echocardiogram and/or ≥25 mmHg by right catheterization), ischemic lesions in fingers (pitting scars, digital ulcers, ES amputations) and dysphagia was also recorded. Specific autoantibodies (anti topoisomerase I, Scl 70; anti centromere; ACA) were determined, and overlapping with other connective tissue diseases was recorded according to expert diagnosis and to classification criteria at the date of this study.

Treatment at the time of inclusion in the study was recorded, as follows: calcium blockers (CCBs), phosphodiesterase 5 inhibitors (IPED5), endothelin antagonists, pentoxifylline, cilostazol, prostaglandin analogs, proton pump inhibitors, prokinetics (domperidone, cisapride/mosapride), glucocorticoids, methotrexate (MTX), mycophenolate mofetil, azathioprine, cyclophosphamide, D-penicillamine, human immunoglobulin, and biologic DMARs (TNF inhibitors, abatacept, tocilizumab, and rituximab).

### Galectin-1 and Galectin-3 Determinations

Serum samples were aliquoted to minimize damage due to freezing and thawing, and stored frozen at –20°C at the Rheumatology Service, Hospital de Clínicas “José de San Martín”. Serum Gal-1 was determined using an in-house ELISA as described (Croci et al., 2012). Serum Gal-3 level was determined using a human Gal-3 ELISA kit (R&D Systems; DY1154), following manufacturer´s instructions.

### Statistical Analysis

Categorical variables, expressed as frequency and percentage, were analyzed with the chi-square test or Fischer's exact test, as appropriate. Continuous variables were expressed as median and interquartile range or mean and standard deviation, according to their distribution, and were analyzed with Student's *t* test, ANOVA or Mann Whitney or Kruskal Wallis *U* test, as appropriate. Correlation tests were performed by Spearman's test and multiple linear regressions. A value of *p* < 0.05 was considered significant.

## Results

From a total of 83 patients, 95% (79/83) were women, with a median (m) age of 58 years (IQR 47–66 years), a median time of evolution of SSc of 5 years (IQR 1–10 years) and a follow-up time of 22 months (IQR 0–63 months). Regarding the clinical variants, 77% of patients (64/83) presented lcSSc, 21% (17/83) dcSSc and 2% (2/83) sineSSc. In addition, 23% (19/83) of patients were classified as overlap syndrome, because of the clinical overlap with other autoimmune diseases (one or more entities): 10 Sjögren's syndrome, 6 Systemic lupus erythematosus, 4 Antiphospholipid syndrome, 2 Primary biliary cholangitis and 5 others (2 autoimmune inflammatory myopathy, 1 ANCA-associated vasculitis, 1 Rheumatoid arthritis, 1 Celiac disease). Clinical features and frequency of autoantibodies are listed in Table 1.

**TABLE 1:** Clinical Manifestations and Autoantibodies Levels in SSc Patients

Clinical Manifestations	Total	lcSSc	dcSSc	ssSSc
	*n* = 83	*n* = 64	*n* = 17	*n* = 2
Raynaud	82 (99)	64 (100)	17 (100)	1 (50)
Skin Fibrosis	72 (87)	55 (86)	17 (100)	0 (0)
Puffy fingers/hands	37 (45)	31 (48)	6 (35)	0 (0)
Microstomy	19 (23)	12 (19)	7 (43)	0 (0)
Tendinous retractions	48 (58)	23 (36)	15 (93)	0 (0)
Telangiectasia	62 (75)	49 (76)	12 (70)	1 (50)
Calcinosis	20 (24)	15 (23)	5 (29)	0 (0)
Pitting scars	30 (36)	18 (28)	12 (70)	0 (0)
Digital ulcers	22 (26)	14 (22)	8 (47)	0 (0)
Digital amputation	6 (7)	4 (6)	2 (12)	0 (0)
Disphagia	31 (37)	23 (36)	7 (41)	1 (50)
Diarrhea	15 (18)	13 (20)	2 (12)	0 (0)
Constipation	22 (26)	19 (29)	3 (18)	0 (0)
ILD	28 (34)	18 (28)	9 (53)	1 (50)
PHT	18 (22)	13 (20)	3 (18)	2 (100)
Arthritis	11 (13)	7 (11)	3 (18)	1 (50)
Muscular weakness	5 (6)	3 (5)	1 (6)	1 (50)
Gastroesophageal reflux	36 (43)	26 (40)	9 (53)	1 (50)
**Autoantibodies**				
ANA	82 (99)	63 (98)	17 (100)	2 (100)
ACA	44 (53)	42 (66)	2 (12)	0 (0)
Scl-70	20 (24)	8 (12)	11 (65)	1 (50)

Data are expressed as n (%). ILD, Intersticial Lung Disease; PHT, Pulmonary Hypertension. ANA, Antinuclear antibodies; ACA, anticentromere antibodies; Scl 70, anti topoisomerase 1.

**FIGURE 1:**
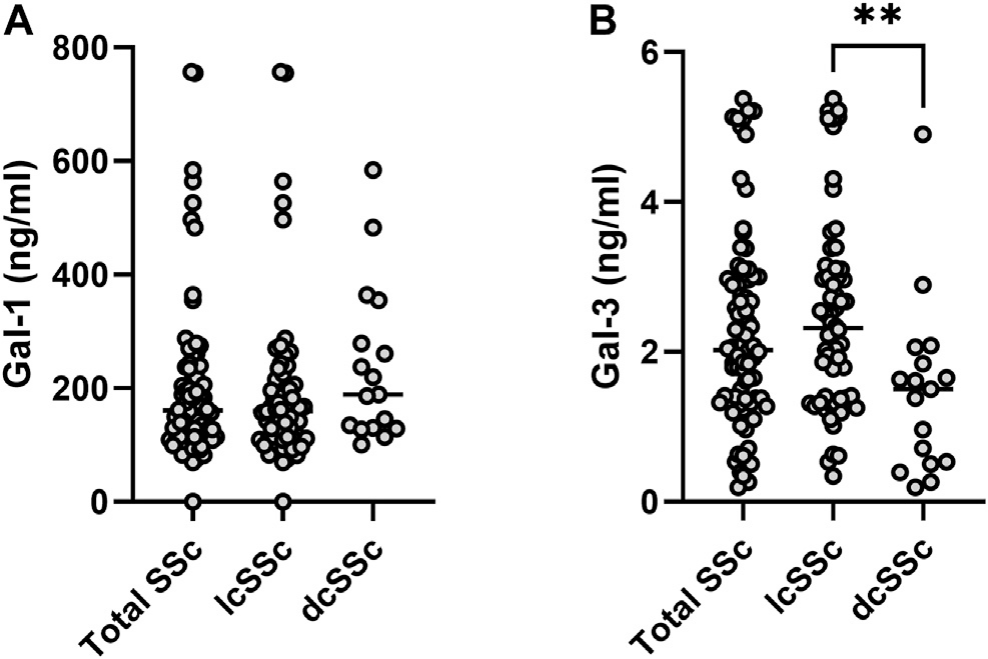
Galectin concentrations in sera from patients with systemic sclerosis (SSc). Upper panel, Gal-1; lower panel, Gal-3. lcSSc, limited cutaneous SSc (*n* = 64); dcSSc, diffuse cutaneous SSc (*n* = 17). Mann-Whitney *U* test was used to compare galectin levels between dcSSc and lcSSc patients. **p < 0.01.

When analyzing the whole population serum samples, Gal-1 concentration was m 162.27 ng/ml (IQR 114.65–235.20 ng/ml) and Gal-3 concentration was m 2.02 ng/ml (IQR 1.31–2.97 ng/ml). When considering gender, women presented higher concentration of Gal-1 (m 166 ng/ml [IQR 117.93–237.93 ng/ml]) than men (m 104.96 ng/ml [IQR 23.87–129.89 ng/ml]) (*p* = 0.019). However, there was no considerable difference in Gal-3 concentrations between men and women (m 2.02 ng/ml [ICR 1.32–2.97 ng/ml] vs. 1.36 ng/ml [ICR 0.26–4.46 ng/ml]) (*p* = 0.09). When stratifying by clinical type of SSc (Figure 1), patients with lcSSc presented a median of 160 ng/ml (IQR 112.85–213.51 ng/ml) for Gal-1, and patients with dcSSc showed a median of 188.78 ng/ml (IQR 129.74–317.25 ng/ml) (*p* = 0.13). Regarding Gal-3, concentrations were 2.32 ng/ml (IQR 1.37–3.08 ng/ml) and 1.50 ng/ml (IQR 0.51–1.95 ng/ml) in patients with lcSSc and dcSSc respectively (*p* = 0.003). Patients with overlap syndrome had a median Gal-1 of 142.84 ng/ml (IQR 114.65–206.96 ng/ml) and Gal-3 of 2.00 ng/ml (IQR 1.38–2.89 ng/ml). Differences in Gal-1 and Gal-3 levels between patients with and without overlap were not significant (*p* = 0.41 and *p* = 0.84, respectively). We found no correlation between the time of evolution measured in years and the concentrations of Gal-1 and Gal-3 (rho 0.05 and 0.02 respectively).

To further investigate the association of serum Gal-1 and Gal-3 with clinical manifestations of SSc, patients were classified into 2 groups according to the presence or absence of organ involvement, and galectin concentrations were assessed in these two groups (Table 2). We found a statistically significant association between higher values of Gal-1 and the presence of telangiectasias (234 ng/ml vs. 157.68 ng/ml, *p* = 0.015) and between higher Gal-3 values and the presence of telangiectasias (2.45 ng/ml vs. 1.69 ng/ml, *p* = 0.021), diarrhea (2.89 ng/ml vs. 2.12 ng/ml, *p* = 0.039) and constipation (2.75 ng/ml vs. 2.08 ng/ml, *p* = 0.038) (Table 2). On the contrary, we found a statistically significant association between lower Gal-3 levels and the presence of tendinous retractions (1.84 ng/ml vs. 2.63 ng/ml, *p* = 0.005). Since higher concentrations of Gal-3 were found in patients with lcSSc, and gastrointestinal manifestations and telangiectasias were more frequent in this clinical form of SSc (Table 1), the association of these clinical manifestations with the lcSSc clinical variant was evaluated. Data revealed no statistically significant association between these clinical parameters. Likewise, given the lower concentration of Gal-3 in patients with dcSSc, we evaluated the association of tendon retractions with this clinical form of the disease. We found a statistically significant association between Gal-3 and tendon retractions in dcSCC patients (*p* = 0.005).

**TABLE 2:** Clinical features and galectin concentrations in SSc patients (*n* = 83)

Clinical signs and symptoms	Presence/ absence	N	Gal-1 ng/ml	p*	Gal-3 ng/ml	p*
Skin fibrosis	(+)	72	218.48	0.64	2.20	0.29
	(–)	11	189.91		2.65	
Puffy fingers/hands	(+)	37	196.99	0.44	2.36	0.52
	(–)	46	228.93		2.17	
Microstomy	(+)	19	308.00	0.08	2.02	0.34
	(–)	64	185.07		2.33	
Tendinous retractions	(+)	48	208.69	0.77	1.84	0.005
	(–)	35	220.53		2.63	
Telangiectasias	(+)	62	234.00	0.015	2.45	0.021
	(–)	21	157.68		1.69	
Calcinosis	(+)	20	180.35	0.35	2.14	0.63
	(–)	63	225.59		2.29	
Pitting scars	(+)	30	217.87	0.90	1.92	0.07
	(–)	53	212.89		2.45	
Digital ulcers	(+)	22	199.99	0.67	2.10	0.50
	(–)	61	219.99		2.31	
Digital Amputation	(+)	6	253.14	0.60	1.57	0.18
	(–)	77	211.70		2.31	
Dysphagia	(+)	31	218.84	0.87	2.36	0.58
	(–)	52	212.22		2.20	
Gastroesophageal reflux	(+)	36	189.05	0.26	2.27	0.93
	(–)	47	235.31		2.25	
Diarrhea	(+)	15	199.00	0.72	2.89	0.039
	(–)	68	218.15		2.12	
Constipation	(+)	22	220.59	0.86	2.75	0.038
	(–)	61	212.56		2.08	
ILD	(+)	28	277.15	0.08	2.16	0.63
	(–)	55	182.90		2.31	
PHT	(+)	18	224.28	0.80	2.16	0.71
	(–)	65	212.04		2.28	
Arthritis	(+)	11	163.02	0.33	2.37	0.75
	(–)	72	222.59		2.24	
Muscular weakness	(+)	5	157.34	0.48	1.32	0.09
	(–)	78	218.37		2.32	

*p-value obtained from comparing Gal-1 or Gal-3 levels from patients presenting or not each clinical manifestation (Mann-Whitney U test). ILD, Interstitial lung disease; PHT, Pulmonary hypertension.

We then evaluated the association of galectins concentrations and the presence of SSc-specific autoantibodies. ACA was present in 53% of patients and Scl-70 in 24%. No association was found between Gal-1 and ACA levels (201.40 ng/ml vs. 229.69 ng/ml, *p* = 0.49) or between Gal-1 and Scl-70 (235.03 ng/ml vs. 208, 23 ng/ml, *p* = 0.058). Similarly, we did not find association between Gal-3 and ACA (2.50 ng/ml vs. 1.98 ng/ml, *p* = 0.07), nor between Gal-3 and Scl-70 (2.03 ng/ml vs. 2.33 ng/ml, *p* = 0.36).

We then analyzed possible relationships between Gal-1 and/or Gal-3 serum concentrations and patient treatment at time of determinations. We found lower concentrations of Gal-3 in patients receiving calcium blockers (2.05 ng/ml vs. 2.64 ng/ml, *p* = 0.048) and in patients receiving MTX (1.54 ng/ml vs. 2.37 ng/ml, *p* = 0.046) compared to those not receiving such treatments (Table 3). In addition, when considering all immunosuppressive treatments (IT) together and grouped patients according to this variable, we found significantly lower concentrations of Gal-1 in dcSSc patients receiving IT (171.32 ng/ml vs. 332.84 ng/ml, *p* = 0.02), and decreased levels of Gal-3 in total SSc patients receiving IT (1.74 ng/ml vs. 2.46 ng/ml, *p* = 0.044) compared to patients not receiving this treatment (Table 4).

**TABLE 3:** Pharmacological treatments in SSc patients studied

Drug	CCB	PDE5-I	ERAs	Cilostazol	Pentoxifylline	PGA	PPIs	Prokinetics
Total SSc	54 (65)	24 (29)	2 (2.4)	12 (14.5)	9 (10.8)	0 (0) 58	(69.9)	5 (6)
dcSSc	14 (82)	7 (41)	1 (6)	2 (12)	3 (18)	0 (0)	11 (65)	0 (0)
lcSSc	40 (62)	16 (25)	0 (0)	10 (16)	6 (9)	0 (0)	45 (70)	5 (8)
**Drug**	**GC**	**MTX**	**MFM/MFS**	**CYC**	**Azathiopine**	**D-Pen**	**Ig IV**	**bDMARs**
Total SSc	11 (13)	11 (13)	3 (3.6)	1 (1.2)	5 (6)	4 (4.8)	0 (0)	0 (0)
dcSSc	4 (23)	6 (35)	2 (12)	1 (6)	1 (6)	1 (6)	0 (0)	0 (0)
lcSSc	6 (9)	5 (8)	1 (2)	0 (0)	3 (5)	3 (5)	0 (0)	0 (0)

Data are expressed as n (%). The numbers of dcSSC + lcSSC may differ from total SSc as patients with ssSSc (n=2) are not shown. CCB, calcium channel blockers; PDE5-I, phosphodiesterase 5 inhibitors; ERAs, endothelin receptor antagonists; PGA, prostaglandin analogs; PPIs, proton pump inhibitors; GC, glucocorticoids; MTX, methotrexate; MFM, mycophenolate mofetil/MFS, mycophenolate sodium; CYC, cyclophosphamide; D-Pen, D-penicillamine; IV Ig, intravenous immunoglobulins; bDMARS, biological disease-modifying antirheumatic drugs. IT, immunosuppresive treatment.

**TABLE 4:** Galectin concentrations in SSc patients with our without immunosuppresive treatment (IT).

Patients	All (*n* = 83)	dcSSc (*n* = 17)	lcSSc (*n* = 64)
Receiving IT	23 (27.7)	10 (58.8)	12 (18.7)
Mean Gal-1 on IT	183.83 ± 139.32	171.32 ± 73.29	193.42 ± 180.27
Mean Gal-1 off IT	228.21 ± 202.16	332.84 ± 143.88	214.39 ± 204.74
	*p* = 0.35	*p* = 0.02	*p* = 0.67
Mean Gal-3 on IT	1.74±0.90	1.47 ± 0.77	2.00 ± 0.95
Mean Gal-3 off IT	2.46 ± 1.38	1.55 ± 1.49	2.59 ± 1.31
	*p* = 0.045	*p* = 0.81	*p* = 0.22

Data are expressed as *n* (%). The numbers of dcSSC + lcSSC may differ from total SSc as patients with ssSSc (*n* = 2) are not shown. CCB, calcium channel blockers; PDE5-I, phosphodiesterase 5 inhibitors; ERAs, endothelin receptor antagonists; PGA, prostaglandin analogs; PPIs, proton pump inhibitors; GC, glucocorticoids; MTX, methotrexate; MFM, mycophenolate mofetil/MFS, mycophenolate sodium; CYC, cyclophosphamide; D-Pen, D-penicillamine; IV Ig, intravenous immunoglobulins; bDMARS, biological disease-modifying antirheumatic drugs. IT, immunosuppresive treatment.

Although there has been no compensation for multiple testing, in multiple linear regression models the presence of telangiectasia and the type of SSc maintained their statistical association with Gal-3 concentrations (*β* 0.25; *p* = 0.022 and *β* 0.26; *p* = 0.017, respectively). However, the association between clinical manifestations of disease and Gal-1 concentrations did not reach statistical significance.

## Discussion

Systemic sclerosis, though an uncommon disease, has a high morbidity and mortality rates. Despite significant efforts in developing new classification criteria to improve earlier diagnosis, none of the proposed systems reflect adequately the heterogeneity of clinical manifestations of SSc (Denton and Khanna, 2017). Thus, an improved understanding of the pathological mechanisms underlying SSc will enable a better management of the disease, including improved classification and more systematic assessment and follow-up.

Compelling evidence suggesting the involvement of galectins in the control of immune and vascular programs, as well as in fibrotic processes support a role for these proteins in SSc. Nevertheless, our current understanding on the impact of galectins in SSc and their clinical relevance remains elusive. Here, we demonstrated the presence of lower concentrations of circulating Gal-3 in patients with diffuse compared to localized forms of SSc, whereas no differences in Gal-1 levels were observed between both groups. Also, associations with clinical manifestations and treatment at time of serological determinations were observed.

Regarding a possible association between circulating Gal-1 and the type of SSc, we found no significant differences between patients with diffuse and limited cutaneous forms, in agreement with previous findings (Yanaba et al., 2016). Nevertheless, considering that dcSSc patients receiving IT showed significant lower Gal-1 levels, the possibility that any potential statistically significant difference in circulating Gal-1 between lcSSc and dcSSC might be blurred out by high prevalence of IT treatment among dcSSc patients cannot be ruled out. Moreover, with regards to associations with clinical manifestations, we found significantly higher levels of Gal-1 in patients with associated telangiectasias, a phenomenon associated with endothelial dysfunction, although no association was observed with the presence of digital ulcers (DU) and pitting scars, in contrast to previous reports (Yanaba et al., 2016), calling into question the suggested protective role for Gal-1 in the development of digital vasculopathy in SSc (Yanaba et al., 2016). Finally, no association was detected between Gal-1 serum levels and the presence of specific SSc antibodies.

Regarding Gal-3, we found significantly higher levels of this lectin in patients with lcSSc as compared with those transiting the diffuse variant, in line with previous findings that showed serum Gal-3 levels relatively decreased in patients with dcSSc compared with lcSSc (Taniguchi et al., 2012). Strikingly, the opposite outcome was recently reported (Stochmal et al., 2020), whereas no significant differences were reported in a smaller cohort (Koca et al., 2014). As discussed below, disparity in the treatment schemes frequently used in the different cohorts analyzed may explain these discrepancies.

Several reports addressed a possible association between Gal-3 serum levels and clinical manifestations of SSc. In accordance with our findings, no significant differences in Gal-3 were found between patients presenting or not DU or pulmonary vascular involvement (Koca et al., 2014), while higher levels were reported only in SSc patients presenting both clinical signs (Taniguchi et al., 2012). Interestingly, we found no association between serum Gal-3 levels and the presence of cutaneous sclerosis. Despite a relative decrease compared to lcSSc, serum Gal-3 levels were found to correlate with the extent of skin fibrosis in dcSSc (Taniguchi et al., 2012). In lesional skin of SSc patients, however, increased local Gal-3 expression was associated with a higher modified Rodnan's skin score (Mora and Zubieta, 2020), suggesting that heightened local, but not systemic, Gal-3 might be responsible of cutaneous fibrosis. Notably, we found a significant association between lower Gal-3 values and the presence of tendinous retractions. Given that this lectin is highly associated with fibrotic processes, higher levels of Gal-3 were expected in these patients. Nevertheless, the significant associations between tendon retractions and the dcSSc variant described herein, and between lower Gal-3 levels and this clinical form, may provide a possible explanation for this finding.

To our knowledge, no previous study reported an association between higher Gal-3 levels and the presence of telangiectasias. Microvascular alterations, characterized by endothelial cell damage, together with mononuclear cell infiltrates and slowly developing fibrosis, are important features of tissue lesions in SSc. Notwithstanding the resulting adaptive response to hypoxia, a paradoxical increase of both pro-angiogenic and angiostatic factors have been detected in early SSc, leading to defective vascularization (Gabrielli et al., 2009). Accordingly, galectins have been shown to influence endothelial cell compartments affecting vascular remodeling and angiogenesis. This effect could explain, at least in part, the association found between higher concentrations of Gal-1 and Gal-3 and the presence of telangiectasias, regardless of the type of SS. In particular, Gal-1 stimulates the migration and proliferation of endothelial cells (Thijssen et al., 2010; Croci et al., 2012; Bastón et al., 2014; van Beijnum et al., 2016), and glycosylation-dependent binding of this lectin to VEGFR2 preserves angiogenesis even in the absence of VEGF (Croci et al., 2014), while both VEGF-dependent and independent proangiogenic effects have been described for Gal-3 (Funasaka et al., 2014).

We found a lower concentration of Gal-3 in patients receiving MTX compared to those not receiving such treatment. Given that most patients taking this drug belong to the dcSSc group, leading to a statistically significant association between dcSSc and the use of MTX (*p* = 0.008), this finding could eventually explain the reduction in Gal-3 found in dcSSC patients. Moreover, in contrast to reports in patients receiving or not GC, and treated or not with at least one disease-modifying anti-rheumatic drug (DMARD) (Koca et al., 2014), we found a significant association between lower Gal-3 levels and the use of any immunossuppresive treatment. Differences in the DMARD most often used, in the frequency of GC usage, as well as in the cohort size and composition, may explain this discrepancy. Similarly to clinical studies in other rheumatologic diseases (Mendez Huergo et al., 2019), and in view of previous findings demonstrating cell type- and drug-specific control mechanisms of Gal-3 expression (Maldonado et al., 2011; Sundblad et al., 2011; Dabelic et al., 2012), differences in treatment schemes should be considered when comparing reports from distinct cohorts. We also found a lower concentration of Gal-3 in patients receiving calcium blockers; though the association between dcSSc and the use of CCB was not statistically significant, we found a biological trend in these patients (83% in dcSSc vs. 62% in lcSSc) that may eventually help to explain this result.

Notably, women presented significantly higher levels of Gal-1 than men, and in contrast to the reported for general population (Cediel et al., 2021), no differences were found in Gal-3 levels. Though variations in fat mass and hormonal conditions might explain these differences, further studies will help to understand these findings.

In conclusion, despite discrepancies due to the heterogeneity of the studied groups, our results and those involving other patient cohorts suggest that Gal-3 may be associated with clinical manifestations and pathological events relevant to development of SSc. Among them, the presence of telangiectasias, which showed clear statistical significance with Gal-3 levels, deserves special attention as it might reflect the central role of this lectin in vascular remodeling and angiogenesis. Further prospective studies, with standardized inclusion criteria will be necessary to define whether Gal-3 is a prominent biomarker of disease activity and/or severity in SSc.

## Data Availability

The raw data supporting the conclusion of this article will be made available by the authors, without undue reservation.

## References

[B1] Allanore Y. Simms R. Distler O. Trojanowska M. Pope J. Denton C. P. (2015). Systemic sclerosis. Nat. Rev. Dis. Primers 1, 15002. 10.1038/nrdp.2015.2 27189141

[B2] Bastón J. I. Barañao R. I. Ricci A. G. Bilotas M. A. Olivares C. N. Singla J. J. (2014). Targeting galectin-1-induced angiogenesis mitigates the severity of endometriosis. J. Pathol. 234, 329–337. 10.1002/path.4397 24979200

[B3] Cediel G. Codina P. Spitaleri G. Domingo M. Santiago-Vacas E. Lupón J. (2021). Gender-related differences in heart failure biomarkers. Front. Cardiovasc. Med. 7, 617705. 10.3389/fcvm.2020.617705 33469552 PMC7813809

[B4] Cerliani J. P. Blidner A. G. Toscano M. A. Croci D. O. Rabinovich G. A. (2017). Translating the ‘sugar code' into immune and vascular signaling programs. Trends Biochem. Sci. 42, 255–273. 10.1016/j.tibs.2016.11.003 27986367

[B5] Croci D. O. Cerliani J. P. Dalotto-Moreno T. Méndez-Huergo S. P. Mascanfroni I. D. Dergan-Dylon S. (2014). Glycosylation-dependent lectin-receptor interactions preserve angiogenesis in anti-VEGF refractory tumors. Cell 156, 744–758. 10.1016/j.cell.2014.01.043 24529377

[B6] Croci D. O. Salatino M. Rubinstein N. Cerliani J. P. Cavallin L. E. Leung H. J. (2012). Disrupting galectin-1 interactions with N-glycans suppresses hypoxia-driven angiogenesis and tumorigenesis in Kaposi's sarcoma. J. Exp. Med. 209, 1985–2000. 10.1084/jem.20111665 23027923 PMC3478924

[B7] Dabelic S. Novak R. Goreta S. S. Dumic J. (2012). Galectin-3 expression in response to LPS, immunomodulatory drugs and exogenously added galectin-3 in monocyte-like THP-1 cells. In Vitro Cell.Dev.Biol.-Animal 48, 518–527. 10.1007/s11626-012-9540-x 22893213

[B8] Denton C. P. Khanna D. (2017). Systemic sclerosis. The Lancet 390, 1685–1699. 10.1016/S0140-6736(17)30933-9 28413064

[B9] Elola M. T. Ferragut F. Méndez-Huergo S. P. Croci D. O. Bracalente C. Rabinovich G. A. (2018). Galectins: multitask signaling molecules linking fibroblast, endothelial and immune cell programs in the tumor microenvironment. Cell Immunol. 333, 34–45. 10.1016/j.cellimm.2018.03.008 29602445

[B10] Faludi R. Nagy G. Tőkés-Füzesi M. Kovács K. Czirják L. Komócsi A. (2017). Galectin-3 is an independent predictor of survival in systemic sclerosis. Int. J. Cardiol. 233, 118–124. 10.1016/j.ijcard.2016.12.140 28043664

[B11] Funasaka T. Raz A. Nangia-Makker P. (2014). Galectin-3 in angiogenesis and metastasis. Glycobiology 24, 886–891. 10.1093/glycob/cwu086 25138305 PMC4153760

[B12] Gabrielli A. Avvedimento E. V. Krieg T. (2009). Scleroderma. N. Engl. J. Med. 360, 1989–2003. 10.1056/NEJMra0806188 19420368

[B13] Gruszewska E. Cylwik B. Gińdzieńska-Sieśkiewicz E. Kowal-Bielecka O. Mroczko B. Chrostek L. (2020). Diagnostic power of galectin-3 in rheumatic diseases. Jcm 9, 3312. 10.3390/jcm9103312 PMC7602543 33076422

[B14] Hromádka M. Seidlerová J. Suchý D. Rajdl D. Lhotský J. Ludvík J. (2017). Myocardial fibrosis detected by magnetic resonance in systemic sclerosis patients - relationship with biochemical and echocardiography parameters. Int. J. Cardiol. 249, 448–453. 10.1016/j.ijcard.2017.08.072 28935460

[B15] Koca S. S. Akbas F. Ozgen M. Yolbas S. Ilhan N. Gundogdu B. (2014). Serum galectin-3 level in systemic sclerosis. Clin. Rheumatol. 33, 215–220. 10.1007/s10067-013-2346-8 23912642

[B16] LeRoy E. C. Black C. Fleischmajer R. Jablonska S. Krieg T. Medsger T. A. (1988). Scleroderma (systemic sclerosis): classification, subsets and pathogenesis. J. Rheumatol. 15, 202–205. 3361530

[B17] Li L.-c. Li J. Gao J. (2014). Functions of galectin-3 and its role in fibrotic diseases. J. Pharmacol. Exp. Ther. 351, 336–343. 10.1124/jpet.114.218370 25194021

[B18] Maldonado C. A. Sundblad V. Salatino M. Elia J. García L. N. Leimgruber C. (2011). Cell-type specific regulation of galectin-3 expression by glucocorticoids in lung Clara cells and macrophages. Histol. Histopathol 26, 747–759. 10.14670/HH-26.747 21472689

[B19] Mendez-Huergo S. P. Hockl P. F. Stupirski J. C. Maller S. M. Morosi L. G. Pinto N. A. (2019). Clinical relevance of galectin-1 and galectin-3 in rheumatoid arthritis patients: differential regulation and correlation with disease activity. Front. Immunol. 9, 3057. 10.3389/fimmu.2018.03057 30687310 PMC6333668

[B20] Mora G. F. Zubieta M. R. (2020). Galectin-1 and galectin-3 expression in lesional skin of patients with systemic sclerosis-association with disease severity. J. Clin. Rheumatol. Publish Ahead of Print. 10.1097/RHU.0000000000001367 32501939

[B21] Sciacchitano S. Lavra L. Morgante A. Ulivieri A. Magi F. De Francesco G. (2018). Galectin-3: one molecule for an alphabet of diseases, from A to Z. Ijms 19, 379. 10.3390/ijms19020379 PMC5855601 29373564

[B22] Stochmal A. Czuwara J. Zaremba M. Rudnicka L. (2020). Altered serum level of metabolic and endothelial factors in patients with systemic sclerosis. Arch. Dermatol. Res. 312, 453–458. 10.1007/s00403-019-01993-y 31667578 PMC7306018

[B23] Sundblad V. Croci D. O. Rabinovich G. A. (2011). Regulated expression of galectin-3, a multifunctional glycan-binding protein, in haematopoietic and non-haematopoietic tissues. Histol. Histopathol 26, 247–265. 10.14670/HH-26.247 21154238

[B24] Sundblad V. Morosi L. G. Geffner J. R. Rabinovich G. A. (2017). Galectin-1: a jack-of-all-trades in the resolution of acute and chronic inflammation. J.I. 199, 3721–3730. 10.4049/jimmunol.1701172 29158348

[B25] Taniguchi T. Asano Y. Akamata K. Noda S. Masui Y. Yamada D. (2012). Serum levels of galectin-3: possible association with fibrosis, aberrant angiogenesis, and immune activation in patients with systemic sclerosis. J. Rheumatol. 39, 539–544. 10.3899/jrheum.110755 22247367

[B26] Thijssen V. L. Barkan B. Shoji H. Aries I. M. Mathieu V. Deltour L. (2010). Tumor cells secrete galectin-1 to enhance endothelial cell activity. Cancer Res. 70, 6216–6224. 10.1158/0008-5472.CAN-09-4150 20647324

[B27] van Beijnum J. R. Thijssen V. L. Läppchen T. Wong T. J. Verel I. Engbersen M. (2016). A key role for galectin-1 in sprouting angiogenesis revealed by novel rationally designed antibodies. Int. J. Cancer 139, 824–835. 10.1002/ijc.30131 27062254

[B28] Wermuth P. J. Piera-Velazquez S. Rosenbloom J. Jimenez S. A. (2018). Existing and novel biomarkers for precision medicine in systemic sclerosis. Nat. Rev. Rheumatol. 14, 421–432. 10.1038/s41584-018-0021-9 29789665

[B29] Yanaba K. Asano Y. Akamata K. Noda S. Aozasa N. Taniguchi T. (2016). Circulating galectin-1 concentrations in systemic sclerosis: potential contribution to digital vasculopathy. Int. J. Rheum. Dis. 19, 622–627. 10.1111/1756-185X.12288 24517166

